# The Expanding Role of Omalizumab: From Food Allergy to Drug Desensitization

**DOI:** 10.3390/ijms26167868

**Published:** 2025-08-14

**Authors:** Bernadetta Kosztulska, Magdalena Rydzyńska, Zbigniew Bartuzi, Magdalena Grześk-Kaczyńska, Natalia Ukleja-Sokołowska

**Affiliations:** 1 Clinic of Allergology, Clinical Immunology and Internal Diseases, Jan Biziel University Hospital No. 2 in Bydgoszcz, Ujejskiego 75, 85-168 Bydgoszcz, Poland; bernadetta-kosztulska@wp.pl (B.K.); magdalena_rydzynska@outlook.com (M.R.); magdalenagrzesk@gmail.com (M.G.-K.); 2 Department and Clinic of Allergology, Clinical Immunology and Internal Diseases, Ludwik Rydygier Collegium Medicum in Bydgoszcz, Nicolaus Copernicus University in Torun, 87-100 Torun, Poland; zbartuzi@cm.umk.pl

**Keywords:** allergy, omalizumab, food allergy, food allergy immunotherapy, drug hypersensitivity reactions, drug desensitization

## Abstract

Although omalizumab is currently approved for a limited number of indications—such as asthma, chronic rhinosinusitis with nasal polyps, and chronic spontaneous urticaria—its potential applications are expanding each year. Owing to its diverse and not yet fully elucidated mechanism of action, including effects on both IgE- and non-IgE-mediated hypersensitivity reactions with delayed onset, this monoclonal antibody may be beneficial in a wide range of allergic and non-allergic conditions. To date, numerous clinical trials and case reports have documented the successful off-label use of omalizumab. It appears particularly promising for patients with difficult-to-treat hypersensitivities, such as food and drug allergies, which continue to pose significant challenges in modern allergology. Even though further research is needed to establish clear indications for its use in these contexts, omalizumab holds considerable potential to enhance the outcomes and clinical efficacy of food immunotherapy and drug desensitization protocols. The aim of this review is to present the current and potential future applications of omalizumab as an adjunctive treatment in food allergy therapy and in desensitization protocols for patients with hypersensitivity to selected drugs.

## 1. Introduction

Omalizumab is a recombinant, humanized monoclonal IgG1 kappa antibody that specifically targets human immunoglobulin E (IgE), playing a pivotal role in the treatment of various allergic disorders [1]. It binds selectively to the Cε3 domain of free IgE, thereby preventing its interaction with the high-affinity FcεRI receptors located on the surface of basophils and mast cells. This interaction also downregulates FcεRI expression on basophils, mast cells, and dendritic cells, ultimately leading to the attenuation of type I hypersensitivity reactions [2,3]. Since its approval by the United States Food and Drug Administration (FDA) in 2003 for the treatment of allergic asthma, the therapeutic indications for omalizumab have been progressively expanded [1,4,5,6]. Omalizumab is widely utilized not only in the management of asthma but also in the treatment of chronic spontaneous urticaria (CSU) and chronic rhinosinusitis with nasal polyps [1,7,8,9]. In 2024, FDA approved the use of omalizumab for the treatment of IgE-mediated food allergy in both children and adults (≥1 year of age). This is an adjunctive therapy aimed at reducing the risk of allergic reactions following accidental allergen exposure. Moreover, current therapeutic off-label applications of omalizumab have expanded to include innovative desensitization protocols for food allergies—particularly in patients with a history of food-induced anaphylaxis—as well as drug desensitization strategies for various drug hypersensitivities, including those associated with chemotherapeutic agents [2,5,10,11].

Over the past years, the clinical application of omalizumab has extended beyond its approved indications to encompass a variety of off-label uses. These include allergic rhinitis, food allergy, Kimura’s disease, eosinophilic granulomatosis with polyangiitis (formerly Churg–Strauss syndrome), eosinophilic esophagitis, vernal keratoconjunctivitis, eosinophilic otitis media, contact dermatitis, and systemic mastocytosis [12,13]. It is plausible that additional conditions may benefit from omalizumab treatment, given its mechanism of action. Although numerous studies have investigated its use across these diverse clinical contexts, in some instances—such as hyper-IgE syndrome (HIES)—alternative biologic therapies may offer superior efficacy [12,14]. In many cases—such as allergic rhinitis—the high cost of therapy limits the use of omalizumab [12]. In March 2025, FDA approved the first biosimilar to Xolair: Omlyclo [15]. The ongoing development of the pharmaceutical market, including the introduction of biosimilars, may contribute to reduced treatment costs and broader accessibility of omalizumab across various indications and patient populations. Particularly promising—though still primarily under investigation in clinical trials—are omalizumab-based therapies targeting food allergies and drug hypersensitivity reactions. A summary of registered and potential indications for omalizumab is provided in Table 1 and Table 2.

It is important to note that off-label drug administration has been a subject of controversy over the years. According to the European Medicines Agency (EMA), off-label use refers to “situations where a medical product is intentionally used for a medical purpose not in accordance with the authorized product information [16].” Similarly, the U.S. Food and Drug Administration (FDA) defines off-label drug use as the use of a drug for indications other than those for which it is approved, at a different dose or dosing regimen, or in patient populations not included in the approved labeling [17]. Although off-label use can offer significant benefits for certain patient groups, it carries potential risks, including ethical, clinical, and legal concerns. Such use requires physicians to take particular care, including obtaining informed consent from the patient and explaining all possible medical consequences of the treatment [16,17,18]. The EMA imposes stricter regulations on off-label drug use in pediatric populations [18]. Off-label use is frequently the subject of scientific investigation. Despite the concerns it raises, it is important to recognize that off-label applications may eventually become approved indications through ongoing research and regulatory review, potentially leading to improved clinical outcomes.

**Table 1:** Approved indications for omalizumab [5,10,19,20].

Indication	Regulatory Agencies That Have Approved the Indication of Omalizumab	Remarks
Allergic Asthma	FDA EMA	FDA: Adults and children 6 years and above; positive SPT results or in vitro reactivity to perennial aeroallergen and insufficient symptom control with inhaled corticosteroids. EMA: Adults and children 6 years and above with positive SPT for aeroallergen (e.g., dust mice, pollen), high frequency of symptoms or awakenings at night, severe symptoms and insufficient symptoms control by inhaled corticosteroids and a long-acting inhaled beta2 agonist.
Chronic Rhinosinusitis with Nasal Polyps (CRSwNP)	FDA EMA	FDA and EMA: Adults (18 years and above).
Chronic Spontaneous Urticaria (CSU)	FDA EMA	FDA and EMA: Adults and adolescents 12 years and above, unsuccessful antihistamine treatment.
Food Allergy and Food Related Anaphylaxis	FDA	Approved by FDA in 2024 as adjunctive therapy aimed at reducing the risk of allergic reactions following accidental allergen exposure; in adults and children over 1 year; omalizumab therapy should be combined with an elimination diet.

**Table 2:** Off-label potential indications for omalizumab [2,5,10,11,12,13].

Indication	Remarks	Evidence
IgE-related Respiratory Diseases (Except Asthma)	Allergic rhinitis Allergic bronchopulmonary aspergillosis (ABPA) Aspirin-exacerbated respiratory disease (AERD) Asthma-COPD overlap (ACO)	No exact guidelines available, low quality of evidence (case reports, studies on small research groups).
Skin Diseases (Except CSU)	Atopic dermatitis (AD) Contact dermatitis Bullous pemphigoid (BP)	Numerous studies have analyzed the use of omalizumab in atopic dermatitis; only a few randomized controlled trials are available. Case reports and anecdotal evidence for other skin diseases (such as BP).
Drug hypersensitivity reactions (DHRs)	As a part of desensitization protocol or a solo agent in treatment Reported cases of successful therapy in various DHRs (chemotherapeutics, insulin, NSAIDs, biologic agents and more)	No exact guidelines available, low quality of evidence (case reports, studies on small research groups).
Other Conditions with hypereosinophilia/hyper-IgE/atopic background	Eosinophilic esophagitis Eosinophilic gastroenteritis Eosinophilic otitis media Atopic keratoconjunctivitis Kimura’s disease Eosinophilic granulomatosis with polyangiitis Hyperimmunoglobulin E syndrome (HIES) Systemic mastocytosis Recurrent anaphylaxis	No exact guidelines available, low quality of evidence (case reports, studies on small research groups).

In the context of food allergy and drug hypersensitivity, omalizumab may be used both as an adjunct to immunotherapy and as part of desensitization protocols. It is important to note that, although some studies use the terms “desensitization” and “immunotherapy” interchangeably, they represent distinct concepts. As described by Tanno et al., desensitization is defined as “a method, procedure, or approach used to induce a temporary state of tolerance to a compound responsible for an allergic and/or hypersensitivity reaction” [21]. Notably, desensitization can be applied to induce tolerance in both allergic and non-allergic hypersensitivity reactions [21]. Immunotherapy is a treatment strategy used to modulate the immune system in response to specific conditions, including hypersensitivity reactions. It can influence both the active and passive components of the immune response [21,22]. Its efficacy has been well established in IgE-mediated diseases, particularly respiratory allergies such as allergic rhinitis and asthma caused by allergens like weed pollen [21]. Immunotherapy aims to induce long-term immune tolerance, whereas desensitization typically provides only temporary tolerance and must be repeated with each administration of the culprit drug [22]. Immunotherapy represents a comprehensive approach that can alter the course of various conditions, including allergic diseases (e.g., allergic rhinitis, asthma, atopic dermatitis) and non-allergic conditions such as infectious diseases (via vaccination), cancers (through non-allergic immunotherapy), autoimmune disorders, and post-transplantation scenarios (via immunosuppressive therapy) [21]. Desensitization, by contrast, is most commonly employed in the management of drug allergies, such as hypersensitivity reactions to insulin, penicillin, or chemotherapeutic agents [22].

The aim of this study is to evaluate the potential role of omalizumab in enhancing the treatment of two of the most challenging conditions in contemporary allergology: food allergy and drug hypersensitivity reactions. This literature review is based on studies retrieved from the PubMed database, including both large-scale clinical studies and case reports that describe the use of omalizumab in food immunotherapy and the management of drug hypersensitivity reactions. A search of the PubMed database conducted for the period 2010–2025 yielded 393 results for the phrase “omalizumab food allergy” and 226 results for “omalizumab food allergy immunotherapy” (there was overlap among some of the studies retrieved). From this body of literature, 30 key articles published between 2016 and 2025 were selected for detailed analysis. The inclusion criteria included, among other factors, the study size and relevance to the topic, specifically, the assessment of the efficacy and safety of omalizumab use in immunotherapy for food allergy. Duplicate studies appearing in both search results were excluded. Additionally, a search of the ClinicalTrials.gov database using the term “food allergy, omalizumab,” 32 studies were found, 13 of which have been completed and had their results published. These clinical trials were also included in the analysis. Additionally, a PubMed search using the terms “omalizumab drug desensitization” and “omalizumab hypersensitivity reactions” for the period 2001–2025 provided 156 and 166 results, respectively. While numerous studies, including ongoing clinical trials, have investigated the use of omalizumab in food allergy, the available literature on drug hypersensitivity is predominantly limited to case reports and small studies. Research focusing on adverse events or hypersensitivity reactions to omalizumab itself were excluded from this review.

## 2. Food Allergy—The Role of Omalizumab in Food Desensitization

### 2.1. Biological Treatment of Food Allergy

Food allergy represents a significant challenge for modern society. It is characterized by the occurrence of objective and reproducible symptoms following the ingestion of a food at a dose normally tolerated by healthy individuals. The underlying mechanism may be IgE-mediated, non-IgE-mediated, or mixed. The course of the disease can be potentially life-threatening, and the clinical presentation is highly variable. Current treatment is limited to the elimination of allergenic foods and symptomatic relief in case of allergic reactions. Research is ongoing to develop effective methods for the treatment of food allergy.

To date, numerous studies have investigated the pathophysiology of food allergy. Available evidence indicates that it develops as a result of damage to the intestinal epithelial barrier, which allows allergens to come into contact with immune system cells, particularly dendritic cells. Patients with mutations in the filaggrin gene are particularly susceptible to this impaired intestinal barrier function [23].

Upon allergen exposure, dendritic cells present antigens to T lymphocytes, leading to a dominant Th2-type immune response. Th2 cells secrete cytokines such as IL-4, IL-5, and IL-13, which stimulate B cells to produce allergen-specific IgE antibodies. These IgE antibodies bind to receptors on mast cells and basophils, which upon re-exposure to the allergen become activated and degranulate, releasing inflammatory mediators such as histamine, tryptase, and prostaglandins, thereby triggering allergy symptoms, including anaphylaxis. Insufficient activity of regulatory T cells and disturbances in immune tolerance mechanisms, such as the PD-1 and CTLA-4 (Cytotoxic T-Lymphocyte-Associated Protein 4) pathways, prevent the immune system from suppressing this abnormal response [24,25]. Molecular pathways involved in the development of food allergy have been studied in numerous research, including those using animal models (such as mice model in the LEAP study) [24]. These mechanisms indicate potential therapeutic targets, including IgE blockers (e.g., omalizumab) and inhibitors of IL-4/IL-13 and IL-5 pathways, which may modify the course of the allergic response.

Initial observations regarding the efficacy of omalizumab in food allergy were reported in pediatric patients with coexisting food allergy and allergic asthma who were receiving omalizumab for asthma treatment. It was observed that omalizumab increased the threshold of allergen reactivity, and some patients were able to tolerate full doses of previously allergenic foods [26,27].

The double-blind, randomized, placebo-controlled OUtMATCH trial, conducted in the United States, was designed to evaluate the efficacy of omalizumab in the treatment of food allergy. The study was divided into three phases. The primary aim of the first phase was to assess whether omalizumab could increase the threshold of tolerated food allergen doses, potentially reducing the risk of allergic reactions due to accidental exposure. This phase enrolled a cohort of 180 participants aged 1 to 55 years (including only three adults), all with a history of peanut allergy and allergy to at least two additional foods from the following: cashew, milk, egg, walnut, wheat, and hazelnut (confirmed by positive results of SPT, sIgE and oral food challenges). Participants were randomly assigned to receive either omalizumab or placebo. Omalizumab was administered every 2 or 4 weeks for 16–20 weeks, depending on body weight and baseline IgE levels. Following the treatment period, participants underwent double-blind oral food challenges with gradually increasing doses of peanut protein and two other selected allergens to assess their reaction thresholds. The results demonstrated that omalizumab significantly raised the threshold for allergic reactions: 67% of participants in the treatment group tolerated at least 600 mg of peanut protein without symptoms, compared to only 7% in the placebo group. Similar improvements were observed for other food allergens [10].

Ligelizumab, a humanized IgG1 monoclonal antibody, binds to IgE with an affinity 88 times higher than that of omalizumab [28]. The NCT04984876 study evaluated its efficacy and safety in patients with peanut allergy. A total of 211 participants aged 12 to 55 years were enrolled and randomized into one of five arms: ligelizumab 240 mg, ligelizumab 120 mg, or placebo followed by a crossover to active treatment. The trial was terminated early due to the need to optimize the dosing regimen [29]. A follow-up trial, NCT05678959, includes participants from the previous study and aims to evaluate long-term safety and efficacy. Ligelizumab is being administered every four weeks at doses of 120 or 240 mg for up to three years, followed by a 16-week observation period. The primary outcomes are being assessed via skin prick tests and oral food challenges. Results have not yet been published.

Studies investigating the efficacy of dupilumab—an IgG4 monoclonal antibody targeting IL-4 and IL-13 signaling—have also included patients with food allergy. One study assessed its efficacy and safety in children and adolescents with peanut allergy. A total of 24 participants aged 6 to 17 years received subcutaneous dupilumab every two weeks for 24 weeks. The primary endpoint was the ability to tolerate ≥ 444 mg of peanut protein during a double-blind, placebo-controlled oral food challenge. Only two participants achieved this endpoint [30].

When interpreting the results of the above studies, it is important to note that the majority of participants were pediatric patients with minimal adult representation. Further research is necessary to assess the efficacy of biologic therapies as monotherapy in food allergy, particularly in adult populations.

### 2.2. Immunotherapy in Food Allergy

Allergen-specific immunotherapy induces a range of immunological changes, with the ultimate goal of establishing long-term tolerance to the allergen. One of the key mechanisms involves the conversion of T helper 2 (Th2) lymphocytes into regulatory T cells (Tregs), which express FOXP3 and produce anti-inflammatory cytokines such as IL-10 and TGF-β. Simultaneously, there is an increase in the number of regulatory B cells (Bregs), which also secrete IL-10. Together, these changes contribute to the suppression of the Th2 inflammatory response and reduce allergen reactivity. Additionally, immunotherapy decreases the levels of allergen-specific IgE while promoting the production of allergen-specific IgG4 antibodies. These IgG4 antibodies compete with IgE for allergen binding, thereby attenuating the allergic response. A reduction in the activation and degranulation capacity of mast cells and basophils—key mediators of anaphylaxis—has also been observed [25,31,32,33,34].

To date, only one product has been approved for oral immunotherapy (OIT) in food allergy: Palforzia, indicated for peanut allergy.

In the following paragraphs, we will demonstrate the potential role of omalizumab in the development of food immunotherapy protocols. Numerous studies are currently underway to evaluate the efficacy of various immunotherapy strategies; however, existing evidence remains inconsistent. Notable studies include the PALISADE trial, which assessed the efficacy and safety of oral immunotherapy with AR101 (Palforzia) in patients with peanut allergy; an open-label extension of the PALISADE trial evaluating the long-term safety and efficacy of AR101 over a two-year follow-up period; the PEPITES trial, which assessed the safety and efficacy of epicutaneous immunotherapy using the Viaskin Peanut patch (VP250) in 365 children; and a study conducted by Fleischer et al. in 2012 evaluating the clinical outcomes of sublingual immunotherapy (SLIT) in peanut-allergic patients aged 12 to 37 years [35,36,37,38]. Despite ongoing development and an increasing number of clinical trials, immunotherapy for food allergy continues to exhibit limited efficacy and a considerable risk of adverse events, including severe allergic reactions. Whether administered orally, sublingually, or epicutaneously, these therapies result in partial desensitization in only a subset of patients, with most studies focused primarily on pediatric populations. The frequency of adverse effects limits both acceptance and practical application in routine clinical care. Therefore, there is a clear need for adjunctive strategies to enhance the safety and effectiveness of immunotherapy. Biologic agents may represent a promising avenue in this context.

### 2.3. The Role of Omalizumab in Immunotherapy Protocols for Food Allergy

Numerous clinical trials are currently evaluating the addition of omalizumab to allergen-specific immunotherapy (AIT) regimens with the aim of enhancing both efficacy and safety. A primary focus is on reducing the incidence of acute anaphylactic reactions during and following immunotherapy. These studies encompass a heterogeneous range of food allergens—including nuts, milk, and eggs—as well as patients with multiple food allergies. Protocol variations include differing dosing schedules, frequencies, and durations of omalizumab administration, and no single protocol has yet emerged as clearly superior in terms of efficacy and safety.

One of the first randomized, double-blind, placebo-controlled trials evaluating omalizumab in conjunction with oral immunotherapy (OIT) in cow’s milk allergy was conducted by Wood et al. in children and young adults aged 7–32 years [39]. Fifty-seven participants were randomized to receive either omalizumab or placebo during a milk OIT protocol, followed by a maintenance phase. At month 28, omalizumab was discontinued, and participants underwent an oral food challenge (OFC). Those who passed continued OIT for an additional two months before pausing therapy for two months and repeating the OFC at month 32. At month 28, 88.9% of the omalizumab group and 71.4% of the placebo group passed the OFC. At month 32, sustained tolerance was observed in 48.1% of the omalizumab group versus 35.7% in the placebo group (24 patients from omalizumab group, 20 patients from placebo group, *p* = 0.42). The omalizumab group experienced significantly fewer dosing-related adverse events during escalation (2.1% vs. 16.1%) and no reactions requiring treatment, compared to 3.8% in the placebo cohort [39]. These findings demonstrate that adjunctive omalizumab enhances the safety profile of cow’s milk OIT.

The second phase of the OUtMATCH trial compared omalizumab monotherapy to omalizumab combined with multi-allergen OIT in a cohort of 117 participants. The primary endpoint was the maximum tolerated dose of allergenic protein, assessed by OFC. Thirty-six percent of subjects on omalizumab alone tolerated at least 2000 mg of protein from three food allergens, compared to nineteen percent in the combination group. Furthermore, no serious allergic events occurred in the omalizumab-only arm, whereas 30.5% of patients in the combined arm experienced such events [40]. Contrary to expectations, monotherapy with omalizumab yielded superior results in both efficacy and safety.

Another trial in 48 children aged 4–15 years with multiple food allergies compared omalizumab+OIT to placebo+OIT. The primary outcome was the proportion of participants who passed a double-blind, placebo-controlled OFC involving at least two food allergens. At week 36, 83% of the omalizumab-treated group passed a 2 g protein challenge in two or more allergens, versus 33% in the placebo group. No serious adverse events were reported, and the overall incidence of side effects was lower in the omalizumab arm [41].

A case report described a 17-year-old patient with cow’s milk-induced anaphylaxis who underwent a rapid desensitization protocol. Prior to a two-day desensitization OIT, the patient received omalizumab 600 mg every two weeks for six months. Following a successful initiation phase, the patient was discharged with a daily milk intake plan—90 mL in divided doses, increasing by 10 mL per meal per week up to 300 mL daily. Omalizumab was planned to be discontinued at six months [42]; subsequent outcomes have not been reported.

A systematic review and meta-analysis of 11 randomized trials concluded that omalizumab may be an effective therapeutic option for patients with IgE-mediated food allergy—both as monotherapy and combined with OIT, particularly in cow’s milk allergy [43]. A larger meta-analysis including 36 trials further confirmed that omalizumab, with or without OIT, significantly increases the tolerated dose of food allergens [5]. Results consistently indicate that omalizumab-assisted OIT enables higher allergen desensitization levels in shorter timeframes than OIT alone, with reduced incidence of adverse events—including anaphylaxis—and improved adherence. However, the durability of acquired tolerance following omalizumab discontinuation remains unclear [44].

A study of 41 pediatric patients with cow’s milk and/or egg allergy who underwent omalizumab+OIT showed that the regimen enabled desensitization without severe adverse reactions during therapy—but severe reactions recurred post-omalizumab withdrawal [45].

In the third phase of OUtMATCH, 60 participants were evaluated for food tolerance after omalizumab cessation. Successful desensitization was defined as maintaining a median daily intake of ≥300 mg protein for 12 months, assessed across allergens including peanut, cashew, egg, milk, walnut, wheat, and hazelnut. Milk, egg, and wheat were successfully tolerated by 61–70% of subjects, while peanut and other nuts were tolerated in 38–56%. Some participants experienced anaphylaxis during this period [46].

An ongoing trial is comparing two dosing regimens of omalizumab versus placebo in combination with OIT targeting three concurrent food allergens [47].

The GA^2^LEN panel has endorsed adjunctive omalizumab therapy in IgE-mediated food allergy, noting improved tolerability of OIT and reduction in adverse events [48].

A summary of selected studies analyzing omalizumab treatment in combination with immunotherapy is presented in Table 3.

Although current evidence from clinical trials and meta-analyses is promising, heterogeneity in protocols, small sample sizes, and limited follow-up durations warrant further large-scale, well-designed randomized controlled trials and meta-analyses. These are needed to identify optimal dosing strategies, confirm clinical efficacy, and establish long-term safety profiles of omalizumab in food allergy immunotherapy.

Despite existing limitations, current data suggest that omalizumab enhances the safety profile of allergen immunotherapy, reduces frequency and severity of adverse events, and accelerates food tolerance induction. Adjunctive omalizumab appears to be a promising strategy for the treatment of food allergy. However, there is still insufficient scientific evidence to support the development of proper dosage recommendations and preparation protocols for the use of omalizumab during immunotherapy for food allergies. Further studies are needed to collect accurate data that could inform the creation of guidelines, potentially leading to the inclusion of these indications in the official labeling approved by major regulatory bodies such as FDA or EMA.

## 3. Omalizumab in Drug Hypersensitivity: Current State of Knowledge

Drug hypersensitivity reactions (DHRs) represent a heterogeneous group of allergic and non-allergic responses mediated by various cytokines and effector cells, constituting a significant clinical challenge across multiple medical specialties [49,50,51]. Sousa-Pinto et al., in a systematic review analyzing 53 studies, estimated that approximately 8.3% of patients report drug hypersensitivity reactions [52]. Accurate epidemiological data on DHRs worldwide are likely underestimated and vary depending on study design. For example, studies investigating DHRs during anti-cancer therapies report incidences ranging from 0.5% to 44% [11]. Severe manifestations of DHRs may lead to life-threatening complications, including organ damage—such as liver or kidney failure—and anaphylaxis [50]. The standard management of DHRs involves symptomatic treatment (including antihistamines, corticosteroids and, in severe cases such as Stevens–Johnson Syndrome or DRESS, immunosuppressive agents), as well as allergological diagnostics aimed at identifying the causative drug [51,53,54]. Once the culprit drug is identified, the typical approach includes avoidance of further exposure [49,51,54]. However, in cases where the drug is essential and no suitable alternatives are available, a desensitization protocol may be implemented [11,51,55]. This involves the administration of gradually increasing doses of the culprit drug, starting from extremely low concentrations, with the goal of inducing temporary tolerance [51]. Avoiding first-line therapies in favor of second- or lower-line treatments may compromise therapeutic efficacy, particularly in chemotherapy [11,49,56,57]. Therefore, successful desensitization to culprit drugs can improve clinical outcomes and survival rates in many cases [11]. Omalizumab appears to be a promising agent in reducing the incidence and severity of DHRs, either as premedication or as an adjunct to desensitization protocols—mainly due to its multifunctional mechanisms of action, including downregulation of FcεRI receptors, modulation of basophil and mast cell’s function, and influence on the Th2-dependent cytokine pathways. However, it should be noted that, despite encouraging clinical evidence, the exact mechanism of action of omalizumab in drug hypersensitivity remains incompletely understood [3,11,58]. Desensitization procedures are sometimes discontinued due to hypersensitivity reactions [57,59]. In these cases, omalizumab may serve as an effective adjunctive therapy, enabling the successful completion of the desensitization protocols [56,59,60].

Effective management of drug hypersensitivity reactions (DHRs) requires a thorough understanding of the underlying molecular and immunological mechanisms. Currently, two main categories of DHRs are recognized: immediate reactions, occurring within approximately one hour after administration of the culprit drug, and delayed reactions, with onset more than one hour after exposure [54]. As summarized by Palardy et al., immunological DHRs are classified into four types according to the Gell and Coombs system [50]:

Classification of drug hypersensitivity reactions:

- Non-allergic (pseudoallergy): e.g., red man syndrome following vancomycin administration;
- Type I: immediate hypersensitivity reactions mediated by IgE antibodies interacting with basophils and mast cells; however, IgG-mediated anaphylaxis has also been reported;
- Type II: antibody-dependent cytotoxic reactions involving IgG and IgM antibodies, such as hemolytic anemia induced by penicillin;
- Type III: immune complex-mediated reactions involving IgG, IgM, and IgA antibodies;
- Type IV: T-cell mediated delayed hypersensitivity reactions, including severe manifestations such as toxic epidermal necrolysis (TEN), drug reaction with eosinophilia and systemic symptoms (DRESS), and Stevens–Johnson syndrome [50,54].

The pathogenesis of some drug hypersensitivity reactions remains incompletely understood [58].

In a 2014 study analyzing 716 patients with DHRs, Barenji et al. demonstrated that risk factors for drug allergy include the presence of coexisting allergic diseases (such as allergic asthma or allergic rhinitis) and the use of specific medications, particularly those prescribed for hypertension, including ACE inhibitors, angiotensin II receptor blockers, and beta-blockers, as well as antidepressants [61]. Genetic polymorphism in specific genes (such as polymorphism of CYP3A4 in male patients allergic to beta-lactams) may serve as a predisposing factor for the development of drug hypersensitivity [54]. However, drug intolerance reactions appear to occur more frequently in women, who accounted for 71% of the reported cases in this cohort [61]. A similar gender disparity is observed in adverse drug reactions (ADRs). As noted by Song et al., ADRs are more common in females (62.7%) than in males (24.5%) [62]. This phenomenon is often attributed to the influence of female sex hormones and their role in modulating inflammatory pathways [62].

To date, omalizumab has primarily been used to reduce side effects during desensitization protocols in patients with a history of DHRs [53]. However, there is limited evidence regarding the actual impact of omalizumab on the incidence of DHRs, as well as on optimal treatment parameters such as appropriate dosing [13,53]. Given omalizumab’s mechanism of action—namely, its ability to lower IgE levels and inhibit activation of basophils and mast cells—this biologic agent holds considerable potential to reduce or prevent hypersensitivity reactions in previously sensitized patients, particularly those with IgE-mediated type I immediate reactions, such as anaphylaxis. Nonetheless, since its exact effects on delayed DHRs remain unclear, many potential applications of omalizumab in this context are yet to be determined. The following sections summarize the currently available data on the use of omalizumab in the treatment of various types of DHRs.

### 3.1. Chemotherapeutics

Hypersensitivity to chemotherapeutic agents (CHTs) is the third leading cause of fatal drug-induced anaphylaxis in the United States and the fifth in Europe [58]. Markaman et al. reported that hypersensitivity reactions occur in approximately 12% of patients with gynecological malignancies treated with carboplatin [63]; however, the true incidence may be underestimated, with some reports suggesting rates as high as 33% for drug hypersensitivity reactions (DHRs) [64]. Chemotherapeutic agents such as platinum compounds (carboplatin, cisplatin, oxaliplatin), taxanes (paclitaxel, docetaxel), L-asparaginase, procarbazine, and epipodophyllotoxins (teniposide, etoposide) are known to have a high potential to induce hypersensitivity reactions (HRs) [58]. The majority of HRs following CHT administration are IgE-mediated [58]. Premedication with H2-blockers and steroids may help manage HRs after CHTs; however, in some cases of hypersensitivity, such as those involving platinum salts (e.g., carboplatin), this approach is ineffective [58]. Therefore, administration of omalizumab may be beneficial in patients hypersensitized to CHTs.

In a study conducted by Grover et al., eight patients with allergies to anti-cancer drugs—including five with advanced-stage cancer and three with early-stage disease—were treated with 150 mg of omalizumab as an alternative to standard desensitization protocols [11]. These patients had various malignancies, such as glioblastoma, oligodendroglioma, hepatocellular carcinoma, lung, rectal, and ovarian cancers. Omalizumab was administered two weeks prior to the culprit drug and continued every day before each subsequent infusion for four weeks [11]. Six patients were allergic to chemotherapeutic agents (procarbazine, oxaliplatin, paclitaxel, and temozolomide), while the other two were allergic to anti-cancer immunotherapeutics targeting programmed cell death-1 (PD-1) or programmed cell death-ligand 1 (PD-L1) [11]. As a result of omalizumab treatment, 87.5% (seven of eight) of the patients were able to receive first-line, optimal therapy for their conditions [11]. As previously mentioned, omalizumab may offer benefits for individuals who experience difficulties undergoing desensitization protocols for chemotherapeutic agents. Elberink et al. described the case of a woman with stage III endometrioid ovarian cancer who experienced carboplatin hypersensitivity. Despite an initially positive response, she developed an allergic reaction during a subsequent carboplatin desensitization protocol within the previous three years [11]. She received 300 mg of omalizumab four weeks prior to, and then biweekly before each dose of chemotherapeutic treatment in her fourth cycle of chemotherapy, which was well tolerated [11]. In a study by Vultaggio et al., three women with carboplatin hypersensitivity and ovarian cancer successfully underwent a 16-step desensitization protocol supplemented with omalizumab [64]. Saura et al. further demonstrated that omalizumab facilitates the safe administration of chemotherapeutic agents that had previously caused anaphylaxis [65]. However, there is one documented case of unsuccessful omalizumab therapy in a patient with hypersensitivity reactions to oxaliplatin [66].

### 3.2. ASA and Other NSAIDs

Current research indicates that omalizumab can be used not only in the treatment of asthma associated with nonsteroidal anti-inflammatory drug (NSAID)-exacerbated respiratory disease (N-ERD), but also for managing hypersensitivity to aspirin and other NSAIDs [2,67,68]. A randomized controlled trial conducted by Hayashi et al. demonstrated that omalizumab reduced aspirin hypersensitivity in patients with aspirin-exacerbated respiratory disease (AERD) [2]. All 16 patients exhibited positive aspirin challenge results during the placebo phase, whereas 62.5% (10 of 16 patients) showed negative aspirin challenge results following omalizumab treatment, as measured by urinary concentrations of leukotriene E4 (LTE4) and tetranor-PGDM. Furthermore, four of the six patients with positive aspirin challenge results demonstrated increased aspirin tolerance, with cumulative doses increasing from 90 mg to 210 mg [2].

Quint et al. also noted that omalizumab may be a valuable tool in preventing NSAID hypersensitivity reactions in individuals with asthma, as drug hypersensitivity reactions to NSAIDs are potentially related to mast cell activation. However, studies on the effects of omalizumab on mast cell function remain contradictory [68]. Importantly, due to its favorable safety profile, omalizumab can be administered to various patient populations, including pregnant women. Pérez Rodríguez et al. reported a case of successful aspirin desensitization in a pregnant patient, with no side effects observed [69]. A 36-year-old woman with a history of miscarriage due to preeclampsia was prescribed 100 mg of aspirin by her obstetrician to prevent recurrence of preeclampsia [69]. She had multiple coexisting allergic conditions, including allergic rhinitis triggered by dust mite exposure, asthma, oral mite anaphylaxis, and NSAID-exacerbated respiratory disease characterized by asthma exacerbations and periorbital angioedema [69]. Although she had previously tolerated 100 mg of aspirin well during testing prior to in vitro fertilization, she experienced palpebral angioedema and mild bronchospasm approximately four hours after aspirin administration during pregnancy [69]. Through a desensitization protocol combined with omalizumab treatment, the patient successfully developed tolerance to therapeutic aspirin dosages [69]. Current evidence suggests that omalizumab use during pregnancy is likely safe [12]. The EXPECT study further supports that omalizumab is not associated with congenital defects [70]. However, the clinical success of this desensitization case may have also been influenced by the concurrent administration of montelukast, which is thought to help prevent adverse reactions during desensitization protocols [69]. As noted by Fernandez et al., aspirin desensitization is likely the only drug desensitization protocol in which discontinuation of omalizumab does not lead to a loss of tolerance or the recurrence of allergic symptoms upon exposure to the culprit drug [13]. A limitation of many studies investigating the use of omalizumab in drug hypersensitivity reactions (DHRs) to aspirin and NSAIDs is that most focus primarily on patients with N-ERD and AERD, which may restrict the generalizability of their findings to individuals with hypersensitivity to a single NSAID.

### 3.3. Antibiotics

Hypersensitivity reactions to some antibiotics are a common clinical problem antibiotics (especially beta-lactams) and anticonvulsants are responsible for 75% of all DHRs [54]. To date, data on the successful use of omalizumab in managing hypersensitivity reactions to antibiotics remain limited. Furthermore, distinguishing true IgE-mediated allergic reactions from pseudoallergic reactions—such as Red Man Syndrome, which is associated with rapid infusion of vancomycin—continues to pose a significant challenge in clinical practice [53,54]. These diagnostic complexities hinder the proper design and interpretation of studies investigating antibiotic hypersensitivities. The application of omalizumab in this context is likely to be constrained by several factors, including the high cost of therapy, which may lead clinicians to favor conventional desensitization protocols or to opt for alternative antibiotic agents.

### 3.4. Insulin

Over the years, the replacement of animal-derived insulin with human insulin analogs has led to a tenfold reduction in the incidence of insulin hypersensitivity reactions (IHRs) [71,72,73]. Nonetheless, insulin allergy still affects an estimated 0.1–3% of diabetic patients receiving insulin therapy [55]. Effective management of insulin hypersensitivity is particularly critical in individuals with type 1 diabetes, who cannot be transitioned to non-insulin hypoglycemic agents [64]. Some patients exhibit hypersensitivity reactions only following subcutaneous administration, while intravenous delivery of insulin does not elicit symptoms [73]. IHRs encompass a spectrum of clinical manifestations, ranging from mild symptoms such as pruritus and erythema to severe, life-threatening anaphylaxis. These reactions can be mediated by various immunological mechanisms, including IgE-mediated immediate hypersensitivity (Type I), immune complex-mediated reactions (Type III), and delayed-type hypersensitivity (Type IV), such as leukocytoclastic vasculitis [56,71,74]. The primary causes of insulin hypersensitivity reactions (IHRs) include protamine sulfate, preservatives such as cresol and phenol, and the insulin molecule itself [71,73]. Delayed hypersensitivity reactions—typically occurring 8 to 24 h post-administration—are particularly associated with insulin preparations containing zinc or protamine [74]. Management of insulin allergy involves various strategies, including switching to an alternative insulin formulation or, in patients with type 2 diabetes, transitioning to non-insulin hypoglycemic agents. Additional therapeutic approaches include the use of antihistamines and desensitization protocols, which aim to induce tolerance through the gradual administration of increasing insulin doses (standard, rush, or ultra-rush protocols). Experimental methods such as pancreatic transplantation and treatment with omalizumab have also been explored [73,75]. As noted by Tian et al., insulin pump therapy may facilitate effective desensitization by enabling precise dose titration and continuous subcutaneous insulin infusion [55]. As with other desensitization protocols, the occurrence of hypersensitivity symptoms during the reintroduction of the culprit insulin remains a significant clinical challenge and may contribute to treatment failure. To improve both tolerability and efficacy, the adjunctive use of omalizumab has emerged as a potentially valuable therapeutic option. According to current evidence, several case reports have documented the successful use of omalizumab in managing insulin hypersensitivity in patients with both type 1 and type 2 diabetes [3,59,76,77]. For example, Mishra et al. described a patient with type 2 diabetes and insulin hypersensitivity who was treated successfully with omalizumab (150 mg every four weeks; a total of 16 doses at the time of reporting), resulting in the subsequent tolerance of insulin glulisine and detemir [59]. Notably, this patient exhibited hypersensitivity to multiple insulin analogs and was the first documented case of insulin glargine allergy [59]. It is plausible that omalizumab-assisted desensitization could extend the duration of symptom control in insulin hypersensitivity. In another case, Said et al. reported a successful rapid desensitization protocol over two weeks (preceded by premedication with loratadine), after which the patient was able to tolerate NPH insulin for up to four years post-desensitization [73].

A potential limitation of omalizumab use in the management of insulin hypersensitivity reactions is its possible effect on glucose metabolism. In some individuals, omalizumab has been reported to elevate blood glucose levels and adversely affect glycemic control [41,46]. One proposed mechanism underlying this phenomenon is a paradoxical increase in histamine levels induced by omalizumab, which may contribute to insulin resistance. However, current evidence regarding the direct role of histamine in glucose metabolism remains inconclusive and at times contradictory [78,79].

### 3.5. Other Drugs

Several cases have been reported of successful administration of omalizumab during treatment protocols for hypersensitivity reactions to elosulfase alfa, monoclonal antibodies (e.g., rituximab), vaccines, latex and enzyme replacement therapies [12,60,79,80,81,82,83,84].

Latex allergy occurs in approximately 0.1% to 7.6% of the general population [85,86]. However, this prevalence is higher among individuals with habitual latex exposure, such as healthcare workers—affecting up to 30% of dentists, 25–50% of nurses, and 50% of surgeons [53]. Another at-risk group includes children with spina bifida [85]. Approximately 49% of individuals allergic to latex also exhibit symptoms of food allergy (e.g., to banana, kiwifruit, or avocado), a condition known as latex–fruit syndrome [86].

Management of latex allergy primarily involves latex avoidance and immunotherapy [85,86]. However, immunotherapy remains challenging: subcutaneous immunotherapy (SCIT) is associated with severe adverse reactions such as anaphylaxis, while sublingual immunotherapy (SLIT) has shown limited effectiveness in some cases [85]. A successful 32-week treatment with omalizumab that reduced skin symptoms of latex allergy was reported back in 2004 [83]. Aruanno et al. described a case of an 11-year-old boy with a history of latex allergy who, during asthma treatment with omalizumab, showed negative diagnostic test results for latex. These tests included skin prick tests (SPTs) with latex, a decrease in specific IgE against latex, skin challenges with sterile and non-sterile medical gloves, and nasal and conjunctival challenges with latex extract [82]. Importantly, the patient experienced no symptoms after clinical contact with latex [82]. The main limitation of these studies is the low quality of scientific evidence and the small sample size. However, future research with omalizumab and other biologic therapies may help develop effective and safe treatments for latex allergy [86]. Some studies demonstrate that the use of omalizumab during desensitization protocols is effective in patients with lysosomal storage diseases who experience hypersensitivity reactions to enzyme replacement therapy. To date, three cases of omalizumab use in such pediatric patients have been reported [87].

Radice et al. described a successful desensitization to polyethylene glycol (PEG), a component of the Comirnaty vaccine [80]. In vaccine desensitization protocols, omalizumab and other biologics may play a crucial role, as corticosteroids—which are generally avoided due to their immunosuppressive effects—are limited to H2-blockers as an alternative [80]. Thanks to omalizumab administration, the patient was successfully vaccinated against SARS-CoV-2 [80].

This approach may offer a promising strategy for managing vaccine hypersensitivity reactions, which is especially important given the global decline in vaccination rates.

### 3.6. Severe Drug Eruptions—Omalizumab as an Alternative Treatment for DRESS and TEN

Omalizumab is used in the treatment of asthma and chronic urticaria; however, there is promising, albeit limited, scientific evidence suggesting that in certain cases, its use may be justified in the management of severe drug eruptions, for instance DRESS (Drug Reaction with Eosinophilia and Systemic Symptoms) or Stevens–Johnson syndrome (SJS). Severe cutaneous adverse reactions, including DRESS, SJS, and toxic epidermal necrolysis (TEN), involve pathogenic mechanisms in which cytotoxic CD8+ T lymphocytes, as well as certain pro-inflammatory eosinophil promoting cytokines (such as IL-4, IL-5, IL-9, IL-13), play a role [50,88,89]. Those conditions are connected with quite high mortality estimated to be about >1% [54]. Omalizumab has the potential to modulate Th2 cytokine pathways and influence histamine 1 receptor activity [88,90]. Consequently, its administration may be beneficial in patients with cutaneous manifestations of drug hypersensitivity reactions, such as DRESS and TEN. Although limited, several case reports have described the use of omalizumab in severe drug-induced eruptions. In 2016, Uzun et al. reported the case of 76-year-old patient with TEN who was successfully treated with a combination of systemic corticosteroids and a single 300 mg dose of omalizumab [91]. Ben Said et al. analyzed 14 cases of corticosteroid-resistant DRESS and found that omalizumab administration may be beneficial in managing this syndrome [92]. These findings suggest that biologic agents such as omalizumab may have a role in the treatment of DRESS, either as an adjunct to corticosteroids or as an alternative in corticosteroid-resistant cases [90,92]. To date, however, no official guidelines or indications have been established for the use of biologics in DRESS management [90].

## 4. Summary

Although omalizumab has been available on the pharmaceutical market for over 20 years, new potential indications continue to emerge across various disease states. Its mechanism of action extends beyond binding free IgE and downregulating FcεRI receptors on basophils and mast cells; it may also involve the modulation of cytotoxic T-cells, pro-inflammatory interleukin pathways and transcriptomic alterations [2,3,88,90,93]. However, the full mechanism remains incompletely understood, suggesting that omalizumab could have broader therapeutic potential [22,24,93]. Preliminary evidence indicated that omalizumab may be effective in managing complex and prevalent conditions, which treatment still seems a challenge—such as food allergy and drug hypersensitivity reactions (including improving drug desensitization protocols). Further large-scale studies are warranted to evaluate the clinical utility of omalizumab in these emerging indications.

## Figures and Tables

**Table 3:** Summary of selected studies analyzing the role of omalizumab in food allergy treatment [39,40,43,48].

Study Title	Population Size	Population Age	Treatment Protocols	Primary Endpoints	Outcomes
Wood et al., 2016 [39]	57 patients (after exclusion and withdrawal: study group 27 patients, control: 28 patients).	7–35 years, 7–32 years after applying exclusion criteria and patients’ withdrawal.	Double-blind, placebo controlled trial, patients randomized 1:1, ongoing milk immunotherapy (MOIT) combined with omalizumab (study group) or placebo (control group); MOIT began 2 weeks after a month treatment of omalizumab or placebo.	Double-Blind Placebo Controlled Oral Milk Challenges SPT with milk extract CD63 upregulation in basophil activation test (BAT).	Achieving tolerance with fewer MOIT doses in omalizumab group (median: 198.0 versus 225, *p* = 0.008), shorter escalation phase (median 25.9 versus 30 weeks, *p* = 0.01).
OUtMATCH second phase [40]	117 patients allergic to peanuts and at least two other foods (cashew, milk, egg, walnut, wheat, hazelnut; allergy confirmed by SPT, sIgE and OFCs) were qualified to study.	1–55 years.	Double-blind, placebo controlled trial, patients underwent double-blind multi allergen OIT and placebo omalizumab or omalizumab/placebo OIT.	Tolerance of at least 2000 mg protein (cumulative 4044 mg) for all three foods.	88% omalizumab group and 51% OIT group completed this phase of study. Omalizumab was superior to OIT (success 36% versus 19%, *p* = 0.031).
Nurmatov et al., 2025 [43]	1010 participants.	Not defined precisely (children and adults).	Systematic review and meta-analysis of 11 RCTs, two secondary reports from RCTs and two ongoing clinical trials.	Not applicable	Significant increase in tolerance of allergenic food in groups with omalizumab as monotherapy or omalizumab with OIT compared to control (risk ratio [RR] = 2.035, 299 patients, seven studies) (control treatment-placebo or no intervention).
The GA^2^LEN panel [48]	953 participants.	Not defined precisely (children and adults).	Systematic review and meta-analysis of 36 studies; Assessing guidelines and recommendations for food allergy therapy with omalizumab (OUtMATCH study was also, among others, included in the meta-analysis).	Not applicable	OUtMATCH study presents the highest level of evidence on the efficacy and safety of omalizumab administration in food allergy therapy. There are many other clinical studies, but they are ongoing, and their results have not yet been published and summarized.

## Data Availability

No new data were created or analyzed in this study. Data sharing is not applicable to this article.

## Abbreviations

The following abbreviations are used in this manuscript:

FDA	United States Food and Drug Administration
CSU	Chronic Idiopathic Urticaria
AERD	Aspirin-Exacerbated Respiratory Disease
DHRs	Drug Hypersensitivity Reactions
AIT	Allergen-Specific Immunotherapy
